# Being Eager to Prove Oneself: U-Shaped Relationship between Competence Frustration and Intrinsic Motivation in Another Activity

**DOI:** 10.3389/fpsyg.2017.02123

**Published:** 2017-12-12

**Authors:** Hui Fang, Bin He, Huijian Fu, Liang Meng

**Affiliations:** ^1^School of Management, Guangdong University of Technology, Guangzhou, China; ^2^Laboratory of Managerial Psychology and Behavior, Guangdong University of Technology, Guangzhou, China; ^3^School of Business and Management, Shanghai International Studies University, Shanghai, China; ^4^Laboratory of Applied Brain and Cognitive Sciences, Shanghai International Studies University, Shanghai, China; ^5^Neuromanagement Lab, Zhejiang University, Hangzhou, China

**Keywords:** competence, competence frustration, need restoration, intrinsic motivation, self-determination theory

## Abstract

Competence frustration has been consistently found to undermine one’s intrinsic motivation in the same activity. However, the relationship between competence frustration in a preceding activity and one’s intrinsic motivation in a subsequent one remains unclear. In order to explore this relationship, self-reported data were collected from 617 undergraduate students of a large comprehensive university in southern China, who took varied courses immediately before taking a less-demanding one. Results suggested a U-shaped relationship between students’ competence frustration in a preceding course and intrinsic motivation in a subsequent one. To be specific, for students whose competence frustration reached the inflection point, a restoration process would be activated if the current course would help restore their competence. Importantly, these students’ competence frustration in a preceding course was found to positively predict their intrinsic motivation level in a subsequent course. As far as we are concerned, this is the first study to reveal a potential positive effect of need frustration outside of its primary thwarting context, which complements and extends existing literatures on the dynamics between need frustration and intrinsic motivation.

## Introduction

### Intrinsic Motivation and Self-Determination Theory

Intrinsic motivation is commonly accepted to refer to one’s internal interest, curiousness, perceived challenge and enjoyment when performing an activity (Deci and Ryan, 1985). In order to explain effects of varied social and contextual factors on intrinsic motivation, self-determination theory (SDT), a motivation theory taking a cognitive perspective, was proposed a few decades ago, which has become one of the most influential theories on intrinsic motivation now. SDT conceptualizes that individuals have three basic psychological needs, which are autonomy, competence and relatedness, respectively (Deci and Ryan, 2000). The need of autonomy refers to one’s need to act with a sense of ownership, to feel psychological free and to have choices. While competence is defined as the need to feel effective and mastery, relatedness concerns the need to feel socially connected, loved and cared for by other individuals (Deci and Ryan, 2000).

Self-determination theory aims to explain how social and contextual factors support or frustrate individuals’ thriving through satisfaction or frustration of basic psychological needs. The benefits of basic need satisfaction have been illustrated in studies across nations, cultures and a multitude of fields, such as education, work, sport, and healthcare (Milyavskaya and Koestner, 2011). When it comes to intrinsic motivation, a recent meta-analysis showed that satisfaction of each basic need positively predicts intrinsic motivation (Van Den Broeck et al., 2016). Besides examining positive effects of need satisfaction, recent studies have begun to explore the “darker side” of need frustration or need dissatisfaction (Gunnell et al., 2013; Bartholomew et al., 2014; Costa et al., 2015). While these two terms seem to be similar, they have significant differences. While need frustration always involves low need satisfaction (need dissatisfaction), low need satisfaction does not necessarily involve need frustration. Importantly, unfulfilled need may not correlate with malfunctioning as robustly as frustrated need does (Vansteenkiste and Ryan, 2013). For instance, if plants could not obtain adequate sunshine or water (i.e., low need satisfaction), they might fail to grow and die over time. However, if salt water was poured to plants (i.e., need frustration), they would wither quickly. In a word, compared with low need satisfaction which may hinder growth, need frustration is more detrimental (Vansteenkiste and Ryan, 2013). Recent studies have demonstrated that need frustration leads to negative outcomes, including ill-being (Bartholomew et al., 2014), job burnout (Gillet et al., 2015a), counter-productive work behavior (Van Den Broeck et al., 2014), cynicism and turnover intentions (Gillet et al., 2015b), and disengagement (Jang et al., 2016). While findings of these pioneering studies are illuminating, most of existing studies only examined the relationship between need frustration and its negative outcomes in the same activity, ignoring the potential long-term impact of need frustration on a subsequent activity. Recently, a group of researchers took the lead to explore the effect of autonomy frustration outside of its primary thwarting context, and it was found to positively predict one’s intrinsic motivation in the subsequent activity (Radel et al., 2014). However, up to now, effects of the frustration of other basic psychological needs, including competence, remain elusive.

Competence frustration refers to the feelings of inadequacy or failure (Bartholomew et al., 2011). The sense of competence wanes in contexts in which challenges are too high, negative feedback is received, and/or the sense of mastery is undermined by person-focused criticism and social comparisons (Ryan and Deci, 2017). When it comes to the educational setting, competence frustration in the classroom was found to be detrimental, and was negatively correlated with vitality while positively correlated with disengagement (Earl et al., 2017). Disengagement is one of the greatest problems that teachers face in classrooms, which accompanies negative classroom conduct and detachment from learning activities (Fredricks, 2014). Every coin has two sides. While some researchers suggested competence frustration to be harmful, other studies suggested that individuals would be better prepared to seek for competence satisfaction when their competence had been frustrated (Sheldon and Gunz, 2009; Radel et al., 2011). According to this line of studies, while competence satisfaction is critical for the maintenance and promotion of intrinsic motivation (Billitz, 2014), competence frustration may also give rise to a motive that pushes an individual into action. Given that autonomy frustration has been found to boost one’s intrinsic motivation in learning the next course (Radel et al., 2014), how students would behave in a subsequent course once their competence had been frustrated beforehand is worth investigating, which is the goal of this study.

### The Restoration Process for Basic Psychological Needs

A predominant view in motivation literatures suggested that the frustration of fundamental needs would lead to a restoration process (Fiske, 2004; Aarts, 2009). In other words, individuals would actively respond to need frustration, with the aim of readjusting their need satisfaction levels. In a pioneering study, the deprivation of autonomy was found to lead to a restoration process, as a controlling context led to the subsequent approaching behavior toward autonomy-related stimuli (Radel et al., 2011). It is worth noting that autonomy-deprived participants were prepared to regain their autonomy only when their perceived competence in the subsequent task was high enough. If perceived competence was low, participants would keep away from autonomy restoration (Radel et al., 2013). In a follow up study conducted in an educational setting, autonomy deprived students were found to show greater intrinsic motivation in the subsequent course if they could exercise autonomy in it (Radel et al., 2014). To sum up, for human beings, the existence of a restoration process for thwarted autonomy has been well-established in previous literatures.

Besides autonomy, experiencing competence is also important for one’s optimal functioning. As a consequence, it is unlikely that an individual would passively accept competence frustration without activating a restoration process. While the restoration process of competence frustration has not been systematically examined, previous studies have begun to observe that competence satisfaction is negatively associated with the desire to experience competence-fulfilling situation. Interestingly, participants whose competence was less satisfied were found to be more likely to strive for competence-enhancing experiences (Sheldon and Gunz, 2009). Based on findings of existing studies, we suggested that one strategy to be adopted to restore competence satisfaction could be to engage in another less demanding activity, which would compensate the thwarted competence in a preceding activity. In other words, if an individual is in a state of competence deprivation and comes upon an activity that offers him/her a sense of competence satisfaction, his/her intrinsic motivation in this activity would get enhanced. Thus, we hypothesized that the prior competence frustration might have a paradoxical effect on one’s intrinsic motivation in a subsequent task. By depriving individuals of their perceived competence, it might provide a motivational force that leads them to engage in a subsequent activity with heightened intrinsic motivation if this activity can bring them a sense of competence. In line with this reasoning, in this study, we proposed that experiencing competence frustration in the preceding course might strengthen students’ intrinsic motivation in the subsequent one.

## Materials and Methods

### Participants and Research Design

To test our hypotheses, we carried out a survey study in a real educational setting. Participants were 617 freshmen (307 females, mean age = 18.5 years) from a large comprehensive university in southern China. This study was reviewed and approved by the Internal Review Board of School of Management, Guangdong University of Technology. Participants nested in 11 different classrooms came from 10 different majors. Students voluntarily participated in this study upon invitation from their Chinese modern history (hereafter referred to as history course) teachers. All participants gave oral consent after having the purpose of the study described by the researchers. In Chinese universities, history is a public-based compulsory course for college students. Compared with other courses, it is much easier to understand for all students. In this university, history courses took place once a week for a whole semester. Moreover, it was preceded by another course for all the participants (this preceding course may vary for students from different classes), as the teaching schedule had been planned ahead before the semester started. Participants were asked to fill in a questionnaire regarding their competence frustration and intrinsic motivation in both the history course and its preceding course. Collected data demonstrated that competence frustration in the history course is significantly lower compared with its preceding course (*p* = 0.001).

### Procedure

In Chinese universities, courses of undergraduates (including history and its preceding course) include two consecutive sessions. This study took place before the start of the first history session in the middle of the semester. Eight history teachers were contacted to help organize this study. Following their agreement and support, the paper questionnaires were handed out to students who voluntarily participated in this study. Before the study formally started, the researchers briefly introduced the questionnaire. Students were told that their teachers would not have any knowledge of their answers, and were emphasized the importance to respond according to their true feelings. The questionnaire consisted of two parts. One part asked questions about the students’ experience in the history course and the other part measured their experience in the course that preceded history. The order of distributed questionnaires was counterbalanced so that half of the participants answered items on the history course first, while the rest participants firstly filled out items regarding the preceding course. The questionnaire took about 3 min to fill out and was completely anonymous. It should be pointed out that the research assumptions were not communicated to these teachers so that they could not convey any expectation to their students. The adopted questionnaire can be found in Supplementary Table 1. Completed questionnaires were collected by the researchers directly.

### Measurement

Intrinsic motivation was assessed with the Interest/Enjoyment subscale of the Intrinsic Motivation Inventory (IMI; Mcauley et al., 1989). The six original items were modified to assess the students’ intrinsic motivation toward the target course (e.g., “I really like taking this course.”; “This course is one of my favorite subjects.”). Participants were asked to rate on a seven-point scale ranging from 1 (Do not fully agree) to 7 (Totally agree). The modified items demonstrated high internal consistency (α = 0.89 for the history course and α = 0.89 for the preceding one).

We measured one’s perception of competence frustration by adapting the basic psychological need satisfaction and frustration scale – work domain (Chen et al., 2015; Schultz et al., 2015). Three items were adapted to assess the students’ perception of competence frustration toward the target course (e.g., “When I am attending this class, I have serious doubts about whether I can learn things well.”). Answers were given on a seven-point scale ranging from 1 (Do not fully agree) to 7 (Totally agree). The three items demonstrated high internal consistency (α = 0.91 for the history course and α = 0.9 for the preceding course).

## Analyses and Results

Because adopting individual as the unit of analysis when there is a hierarchically nested design (i.e., students nested into classrooms) may influence the results (Kashy and Kenny, 2000), it is necessary to first check whether there are significant between-group differences. If there were significant between-class differences, then a hierarchical linear model (HLM) analysis would be appropriate. Intra-class correlation coefficient (ICC) was adopted to quantify the degree of similarity among classes. Results suggested that there were no significant between-group differences. Thus, there is no need to conduct a cross-level analysis (James et al., 1984).

To examine the effect of competence frustration on one’s intrinsic motivation in the same course, for both the history course and its preceding course, one’s intrinsic motivation was regressed on the level of competence frustration, the presentation order of the questionnaire, class, and major. Results from regression analyses showed that competence frustration was negatively correlated with intrinsic motivation in the preceding course of history (β_1_ = -0.472, *p* < 0.001), and that competence frustration accounted for 23.2% of the total variance. For the history course, competence frustration was negatively correlated with intrinsic motivation (β_1_ = -0.351, *p* < 0.001), and competence frustration accounted for 16.7% of the total variance. Effects of the presentation order of the questionnaire, class, and major on intrinsic motivation did not achieve significance (*p* > 0.05). Taken together, these findings indicated that competence frustration negatively predicted one’s intrinsic motivation in the same course.

Based on the actual scatterplot depicting the relationship between competence frustration in the preceding course and intrinsic motivation in current course (see Supplementary Figure 1), we constructed a quadratic regression to explore the effect of competence frustration in the preceding course (PCF) on one’s intrinsic motivation in the subsequent course (the history course):

$$Y=\beta_{0}+\beta_{1}PCF+\beta_{2}\left(PCF*PCF\right)$$

**FIGURE 1:**
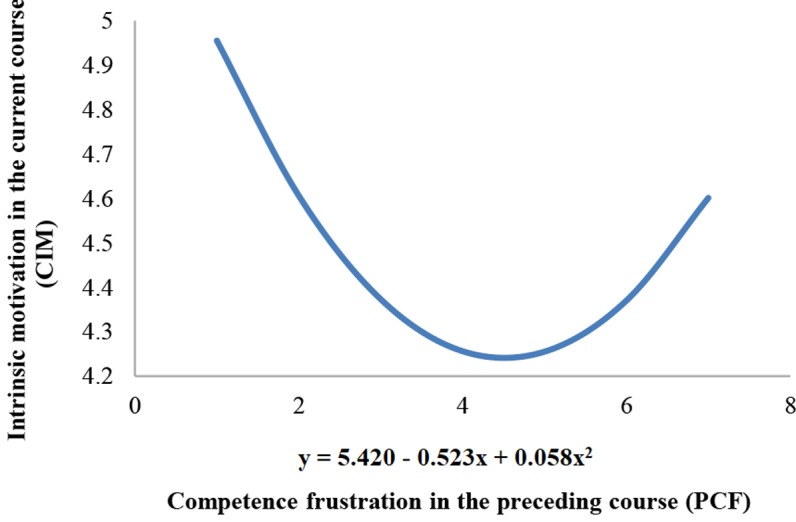
U-shaped curvilinear relationship between one’s competence frustration in the preceding course (PCF) and intrinsic motivation in the current course (CIM).

Regression analysis showed that β_1_ = -0.523, *p* < 0.001; β_2_ = 0.058, *p* < 0.01. Interestingly, we found that there existed a U-shaped curvilinear relationship between competence frustration in the preceding course and intrinsic motivation in the history course, which was not exactly in line with our prediction. From **Figure 1**, we can see that there was an inflection point. Before that point, competence frustration in the preceding course was negatively correlated with one’s intrinsic motivation in history. Beyond that point, it was positively correlated with one’s intrinsic motivation in the history course instead. According to the calculation, the value of the inflection point is 4.7.

**Table 1** presents the means, standard deviations, and internal reliability coefficients of research variables, as well as correlations between the variables when PCF > 4.7. According to the data, competence frustration in the preceding course was positively correlated with intrinsic motivation in the history course (*r* = 0.254, *p* < 0.05), while competence frustration in the history course was negatively correlated with intrinsic motivation in the same course (*r* = -0.273, *p* < 0.05). This provides the necessary prerequisites for subsequent analysis.

**Table 1:** Means, standard deviations, and correlations between research variables (PCF > 4.7).

Variables	*M*	*SD*	1	2	3	4
(1) Competence frustration in the preceding course	5.68	0.73	1			
(2) Intrinsic motivation in the preceding course	3.66	1.13	-0.036	1		
(3) Competence frustration in the history course	3.35	1.58	-0.05	0.139	1	
(4) Intrinsic motivation in the history course	4.2	1.2	0.254^∗^	0.194	-0.273^∗^	1


*^∗^*p* < 0.05, ^∗∗^*p* < 0.01, ^∗∗∗^*p* < 0.001. *N* = 78.*

When PCF > 4.7, the level of intrinsic motivation in the history course was regressed on competence frustration in the preceding course, the level of intrinsic motivation in the preceding course, and the perception of competence frustration in the history course. The structure of regression model is based on a previous study which examined the effect of autonomy frustration (Radel et al., 2014). The regression analysis results are shown in **Table 2.** According to **Table 2**, this model was tenable (*F*_3,77_ = 3.133, *p* < 0.01) and accounted for 20% of the total variance. Competence frustration in the history course was negatively associated with intrinsic motivation in the same course (β_3_ = -0.218, *p* < 0.05). Intrinsic motivation in the preceding course was also a vital predictor of intrinsic motivation in the history course (β_2_ = 0.275, *p* < 0.05). More importantly, competence frustration in the preceding course turned out to positively predict one’s intrinsic motivation in the history course (β_1_ = 0.386, *p* < 0.05).

**Table 2:** Regression analysis of the students’ intrinsic motivation in the history course (PCF > 4.7).

Model	Non-standardized coefficient		Standardized coefficient	*T*	Significance
				
	*B*	The standard error	Beta		
Constant	2.227	1.22		1.825	0.072
Major	-0.083	0.13	-0.2	-0.637	0.526
Class	0.003	0.012	0.089	0.28	0.781
Presentation order of the questionnaire	-0.144	0.261	-0.06	-0.553	0.582
Competence frustration in the preceding class	0.386^∗^	0.184	0.235	2.099	0.039
Intrinsic motivation in the preceding course	0.275^∗^	0.118	0.258	2.329	0.023
Competence frustration in the history course	-0.218^∗^	0.083	-0.285	-2.617	0.011


*Dependent variable: intrinsic motivation in the history course; ^∗^*p* < 0.05, ^∗∗^*p* < 0.01, ^∗∗∗^*p* < 0.001. *N* = 78.*

**Table 3** shows the correlation results between research variables when PCF ≤ 4.7. It is worth noting that competence frustration in the preceding course was negatively correlated with intrinsic motivation in history (*r* = -0.171, *p* < 0.01). Regression results are displayed in **Table 4.** It was found that competence frustration in the preceding course did not have a significant impact on intrinsic motivation in the history course (*r* = -0.056, *p* = 0.245). At first glance, results from the correlation analysis and the regression analysis seemed to be in conflict. We suggested that inclusion of additional predictors in the regression analysis may account for the discrepancy in these results. To be specific, we proposed that: when PCF ≤ 4.7, competence frustration in the preceding class may negatively influence intrinsic motivation in the history class through the mediated roles of intrinsic motivation in the preceding class and competence frustration in the history class. We adopted Mplus 6.11 to analyze the mediation effects, and the sizes of the indirect (i.e., mediated) effects are presented in Supplementary Table 2. The hypothesized model showed an acceptable fit to the data, with χ^2^(539) = 333.28, CFI = 1.0, TLI = 1.002, RMSEA = 0.001, and SRMR = 0.012. As shown in Supplementary Table 2, the two indirect effects were significant. Specifically, both competence frustration in the history class (Estimate = -0.173, *p* < 0.001) and intrinsic motivation in the preceding class (Estimate = -0.05, *p* < 0.05) mediated the effect of competence frustration in the preceding class on intrinsic motivation in the history class.

**Table 3:** Means, standard deviations, and correlations among the study variables (PCF ≤ 4.7).

Variables	*M*	*SD*	1	2	3	4
(1) Competence frustration in the preceding course	2.61	1.18	1			
(2) Intrinsic motivation in the preceding course	4.67	1.17	-0.452^∗∗^	1		
(3) Competence frustration in the history course	2.52	1.35	-0.407^∗∗^	0.158^∗∗^	1	
(4) Intrinsic motivation in the history course	4.55	1.2	-0.171^∗∗^	0.155^∗∗^	-0.422^∗∗^	1


*^∗^*p* < 0.05, ^∗∗^*p* < 0.01, ^∗∗∗^*p* < 0.001. *N* = 539.*

**Table 4:** Regression analysis of the students’ intrinsic motivation in the history course (PCF ≤ 4.7).

Model	Non-standardized coefficient		Standardized coefficient	*T*	Significance
				
	*B*	The standard error	Beta		
Constant	4.919	0.338		14.559	0.000
Major	-0.075	0.049	-0.184	-1.519	0.129
Class	0.005	0.004	0.137	1.134	0.257
Presentation order of the questionnaire	0.011	0.094	0.005	0.118	0.906
Competence frustration in the preceding class	0.056	0.048	0.055	1.164	0.245
Intrinsic motivation in the preceding course	0.111^∗^	0.045	0.108	2.471	0.014
Competence frustration in the history course	-0.373^∗∗∗^	0.038	-0.419	-9.763	0.000


*Dependent variable: intrinsic motivation in the history course. ^∗^*p* < 0.05, ^∗∗^*p* < 0.01, ^∗∗∗^*p* < 0.001. *N* = 539.*

## Discussion

As a basic psychological need, competence is critical for the maintenance and promotion of one’s intrinsic motivation (Billitz, 2014). Thus, when faced with competence frustration, individuals may take active actions to restore it. In our daily life, people frequently participate in a series of activities, and their psychological experience in a preceding activity may affect that in the current one. For instance, a recent study conducted in the educational setting suggested that autonomy-frustrated students would show greater intrinsic motivation in a subsequent course if they could exercise autonomy in it and then regain their perceived autonomy (Radel et al., 2014). We predicted that this would also be true for competence, and assumed that individuals would be more intrinsically motivated for a subsequent activity when it followed one in which they experienced greater competence frustration. To examine our hypotheses, we conducted a survey study in an educational context, findings of which provided partial support for our assumptions.

Previous studies consistently reported that when a task thwarts an individual’s need of competence, his/her intrinsic motivation in the task will decline (Ryan and Deci, 2000; Tsai et al., 2008). In line with these findings, we also observed that competence frustration undermined students’ intrinsic motivation in the same course. This result is also consistent with a previous research conducted in the education domain concerning the effect of competence frustration on disengagement (Jang et al., 2016). When it comes to the relationship between competence frustration in a preceding course and intrinsic motivation in a subsequent one, we predicted this effect to be linear, and more specifically, positive. Interestingly, our hypothesis was not fully supported, as we discovered a U-shaped curvilinear relationship between competence frustration in a preceding course and intrinsic motivation in a subsequent one. Before reaching the inflection point, there was no significant direct effect of competence frustration in a preceding course on one’s intrinsic motivation in a subsequent one. Only after reaching the inflection point (when competence frustration in a preceding course is high enough) would the influence of competence frustration be positive. These results provided preliminary evidences for the paradoxical effect of competence frustration in a given activity on one’s intrinsic motivation in a subsequent activity.

Findings of our study complement and extend existing findings on intrinsic motivation. Most of previous studies examined either characteristics of the activity itself or the context of the activity on one’s intrinsic motivation (Dysvik and Kuvaas, 2011; Abuhamdeh et al., 2014; Baranes et al., 2014; Hofferber et al., 2016). This study went a step further as it argued that one’s experience in a preceding activity could also be an important predictor of his/her intrinsic motivation in the current task. Here, we took into account the role of competence frustration. Previously, the impact of competence frustration has mostly been measured and observed immediately (Bartholomew et al., 2014; Earl et al., 2017). This research widened the research scope and explored its potential effect outside of the primary thwarting context. Our results suggested that, the impact of competence frustration could go beyond the context where the threat occurred and further affect individuals’ psychological status in subsequent activities. While findings of this study seemed to suggest that competence frustration could lead to positive outcomes, this conclusion needs further verification and should be utilized with caution. For instance, previous studies have argued that, while compensation for need frustration in another life domain may occur, the total effect (need frustration + compensation) may be less positive than the situation wherein this need has been satisfied in the first place (Milyavskaya et al., 2009; Emery et al., 2015).

A contribution of our findings is that competence as a basic psychological need gets further confirmed, as one was found to actively seek for competence satisfaction when he/she perceived severe competence frustration beforehand. Future research may follow this line of work (Radel et al., 2011, 2013, 2014) to further clarify the need restoration process over time. Besides competence, other psychological needs could well be analyzed in a similar manner. For example, frustrating one’s relatedness in a prior activity may have a similar impact on his/her intrinsic motivation in a subsequent irrelevant activity if the second activity can satisfy one’s relatedness. While results from the manipulation check suggested that the history course was significantly less competence-frustrating compared with its preceding courses, a limitation of this study is that competence frustration was not directly manipulated. Thus, at the current stage, we cannot be conclusive on the causal relationship between competence frustration in the preceding course and the level of intrinsic motivation one displays in the current course. Experimental studies in which competence frustration is directly manipulated by the researchers are required to establish this causal link. Besides, given that the present study only measured intrinsic motivation through self-report, it will be interesting to resort to behavioral measurement and/or neural indicators of intrinsic motivation to further clarify the long-term consequence of competence frustration triggered by the overwhelming challenge, demanding tasks, or negative feedbacks (Meng et al., 2016; Domenico and Ryan, 2017; Ma et al., 2017; Meng and Yang, 2018). Another limitation of this study is that only 78 students satisfied the criteria of PCF > 4.7. Thus, the sample size of the high competence frustration group is relatively small. The fact that difficulty of the curriculum normally matches abilities of general students in the university may partially account for the current situation. Since the instructor may flexibly tailor difficulty of his/her course according to performances and responses of the students, normally few students would report their perceived competence to be frustrated to a great extent. To replicate findings of this study, future studies with larger sample sizes are highly recommended.

Findings of this study may bear important implications for the educational practice. As students’ competence frustration was found to have a negative influence on their intrinsic motivation in the same class, instructors (especially those teaching a difficult course) should pay special attention to protect competence of their students. For instance, they may consider giving timely positive feedbacks whenever possible so as to boost the students’ sense of competence. In addition, because the restoration process for competence frustration exists, when scheduling courses for the next semester, educational administration personnel should try to make sure that a highly difficult and challenging course is to be followed by a relatively easy one. Indeed, scientific and reasonable course arrangement can enhance students’ intrinsic motivation and improve their overall academic achievements.

## Conclusion

Based on a survey study conducted in a real educational setting, it was found that competence frustration would decrease students’ intrinsic motivation in the same activity. Interestingly, if competence frustration in this activity was high enough, which exceeded a critical point, a restoration process would be activated to help individuals regain competence in the subsequent less demanding activity, and participants would show enhanced intrinsic motivation in it. Through exploring effects of the frustration of a basic psychological need (competence frustration) outside of the primary thwarting context, we complement and extend existing studies on the dynamics between need frustration and intrinsic motivation.

## Author Contributions

LM and HF conceived and designed the study. HF collected and analyzed the data. HF and LM interpreted the data and drafted the manuscript. LM, HF, BH, and HjF reviewed and edited the manuscript. LM administered the project.

## Conflict of Interest Statement

The authors declare that the research was conducted in the absence of any commercial or financial relationships that could be construed as a potential conflict of interest.
