# Supplementary material for: Being Eager to Prove Oneself: U-Shaped Relationship between Competence Frustration and Intrinsic Motivation in Another Activity
Source: Front Psychol. 2017 Dec 12;8:2123. doi: 10.3389/fpsyg.2017.02123 (PMC5733033; doi:10.3389/fpsyg.2017.02123)
Supplement: Supplementary file 1 [file Supplementary_Material.pdf]

Supplementary Material

Being Eager to Prove Oneself: U-Shaped Relationship between Competence Frustration and Intrinsic Motivation in Another Activity

Hui Fang, Bin He, Huijian Fu, Liang Meng*

*** Correspondence:** Liang Meng: promise_land@zju.edu.cn

1 Supplementary Figures and Tables

1.1 Supplementary Figures

1.1.1 Supplementary Figure 1

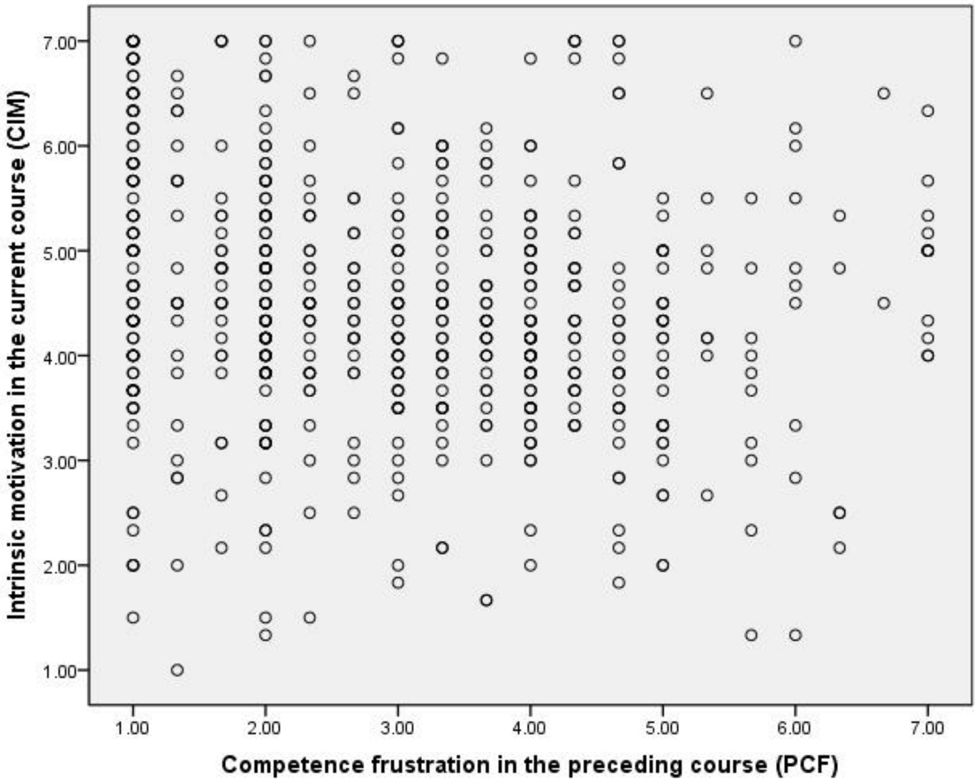

FIGURE 1│The scatterplot depicting the relationship between competence frustration in the preceding course and intrinsic motivation in the current course.

1.2 Supplementary Tables

1.2.1 Supplementary Table 1

TABLE 1 | A Questionnaire about Classroom Experience

This survey is carried out to learn about your impressions on some courses you are taking in this semester. It is completely anonymous and your ratings will not be revealed to the instructors. Please try to answer quickly - usually the first response that comes to mind best reflects your true feelings (your “gut” reaction). Thank you for taking the time to fill in this questionnaire and wish you great success in your future studies.

Part 1: Please draw a "circle" or put a "tick" on the number that best represents your feelings toward the Chinese modern history course.

**Items**	**1: Do not fully agree**			⟶			**7: Totally agree**
1. When I am attending this class, I have serious doubts about whether I can learn things well.	1	2	3	4	5	6	7
2. I feel insecure about my abilities in this class.	1	2	3	4	5	6	7
3. For this course, I expect failure and to feel incompetent.	1	2	3	4	5	6	7
4. I really like taking this course.	1	2	3	4	5	6	7
5. I really get involved in this classroom.	1	2	3	4	5	6	7
6. I think it's a boring course.	1	2	3	4	5	6	7
7. This course is one of my favorite subjects.	1	2	3	4	5	6	7
8. I have fun attending this course.	1	2	3	4	5	6	7
9. This course does not attract my attention at all. What the professor says does not interest me.	1	2	3	4	5	6	7

Part 2: Now, you are asked to fill in the same questionnaire again. However, this time, do not evaluate the Chinese modern history course but the course that precedes it (the course you had just taken before coming to the history class). Please draw a "circle" or put a "tick" on the number that best represents your feelings toward the preceding course.

**Items**	**1: Do not fully agree**			→			**7: Totally agree**
1. When I am attending this class, I have serious doubts about whether I can learn things well.	1	2	3	4	5	6	7
2. I feel insecure about my abilities in this class.	1	2	3	4	5	6	7
3. For this course, I expect failure and to feel incompetent.	1	2	3	4	5	6	7
4. I really like taking this course.	1	2	3	4	5	6	7
5. I really get involved in this classroom.	1	2	3	4	5	6	7
6. I think it's a boring course.	1	2	3	4	5	6	7
7. This course is one of my favorite subjects.	1	2	3	4	5	6	7
8. I have fun attending this course.	1	2	3	4	5	6	7
9. This course does not attract my attention at all. What the professor says does not interest me.	1	2	3	4	5	6	7

Part 3: Basic Information

Sex: ____________

Your major: ____________

Your class: ____________

1.2.2 Supplementary Table 2

TABLE 2 | Mediating Effect Analysis

Indirect Effect	Estimate	SE	90% CI
1.PCF→CCF→CIM	-0.173***	0.023	[-0.22, -0.14]
2.PCF→PIM→CIM	-0.05*	0.025	[-0.05, -0.01]
3.Total Mediation Effect	-0.22***	0.034	[-0.29, -0.17]

Note: Standardized and unstandardized values of indirect effects are highly consistent in this study. Since it is recommended that bootstrapping results be unstandardized (Preacher & Hayes, 2008), unstandardized values are reported. Bootstrapping sample size = 5000; PCF = competence frustration in the preceding course, PIM = intrinsic motivation in the preceding course, CCF = competence frustration in the current course, CIM = intrinsic motivation in the current course. $^{*}p < 0.05$, $^{**}p < 0.01$, $^{***}p < 0.001$. N = 539.
